# Wettability of Kraft Paper with Biobased Impregnation Resins for the Production of High-Pressure Laminates

**DOI:** 10.1021/acsomega.5c03769

**Published:** 2025-08-08

**Authors:** Elisabeth Billich, Elfriede Hogger, Ivan Sumerskii, Wilfried Sailer-Kronlachner, Catherine Rosenfeld, Martina Duller, Christof Blum, Antje Potthast, Hendrikus W.G. van Herwijnen

**Affiliations:** a Wood K plus-Kompetenzzentrum Holz GmbH, Altenberger Strasse 69, Linz 4040, Austria; b Department of Natural Sciences and Sustainable Resources, Institute of Chemistry of Renewable Resources, 27270 BOKU University, Konrad-Lorenz-Strasse 24, Tulln an der Donau 3430, Austria; c Department of Natural Sciences and Sustainable Resources, Institute of Chemistry of Renewable Resources, Core Facility Analysis of Lignocellulosics, 27270 BOKU University, Konrad-Lorenz-Strasse 24, Tulln an der Donau 3430, Austria; d Department of Natural Sciences and Sustainable Resources, Institute of Wood Technology and Renewable Materials, 27270 BOKU University, KonradLorenz-Strasse 24, Tulln an der Donau 3430, Austria

## Abstract

High-pressure laminate (HPL) manufacturers are increasingly looking for more environmentally friendly solutions for their production processes to reduce their environmental footprint. Replacing conventional phenol-formaldehyde resins (PF) with sustainable alternatives from renewable resources still remains a challenge to address. The suitability and wetting behavior of an impregnation resin are strongly dependent on its compatibility with the paper used. Thus, kraft papers with varying recycled fiber content were analyzed regarding their chemical composition, structural morphology, and wettability. Recycled paper exhibited a lower surface free energy and increased two-sidedness compared to paper made from virgin fibers, which was attributed to a higher lignin and extractives content as well as alterations in the fiber morphology due to the recycling process. Additionally, biobased resins derived from carbohydrates and their derivatives were synthesized and evaluated for their wetting behavior. Contact angle measurements and impregnation trials demonstrated distinct differences in penetration behavior between commercial PF resins and biobased alternatives. Despite these differences, the biobased resins exhibited comparable overall wettability, though with different penetration dynamics, highlighting their potential for sustainable HPL production.

## Graphical abstract

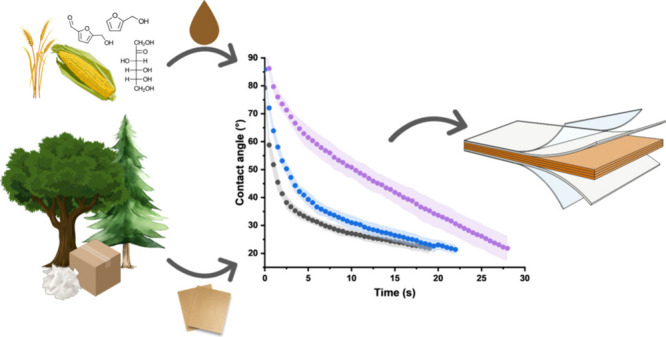

## Introduction

Conventional high-pressure laminates (HPL) consist of several layers of paper treated with impregnation resins (Figure 1). The core of HPL consists of kraft paper impregnated with phenol-formaldehyde (PF) resins, while the top and bottom layers consist of decorative paper impregnated with melamine-formaldehyde (MF) resins.^1,2^ Often, a translucent, MF-impregnated overlay sheet is added to further improve surface properties.^1,3–5^

**Figure 1:**
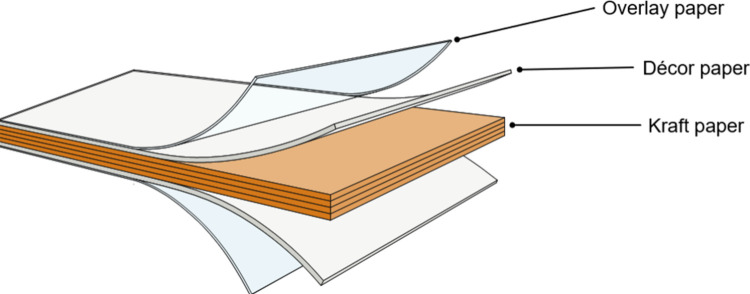
Layered assembly of HPL.

In an industrial impregnation line, paper webs are passed through a resin bath allowing the paper to saturate with the resin. Excess resin is removed by squeezing rolls, and then, the paper is dried before it is cut to the desired dimensions. The impregnated paper sheets are stacked accordingly and cured under high pressure and temperature in a hot press. In this process, the resin flows within and between the paper layers undergoing irreversible cross-linking until they are fully cured. Thus, a very stable resin-paper network is formed providing the final panel with excellent mechanical properties.^2,5^ This allows HPL to be used in a multitude of applications, e.g., furniture production or flooring. Furthermore, the panels are resistant to moisture, chemicals, and fire, making them suitable for demanding use cases, such as in chemistry laboratories or wet rooms, as well as in exterior applications like for balconies and wall claddings.^1^

Although HPL offer numerous advantages, their excellent properties primarily stem from the stability of fossil-based PF resins.^2,6,7^ However, phenol and formaldehyde, the primary components of PF resins, are hazardous chemicals associated with health risks due to their volatile emissions. Additionally, their fossil origin fuels environmental concerns. While the laminate industry is already increasingly focusing on the use of recycled kraft paper to decrease their environmental footprint, the transition to more sustainable impregnation resins from renewable resources would be an additional impactful solution.^8^

To avoid formaldehyde-based formulations, a plethora of studies have investigated the potential of a wide range of renewable resources in the production of adhesives for wood-based materials.^9–18^ In contrast, past research on renewable impregnation resins for HPL has mainly focused on partially substituting phenol with natural phenolic resources, e.g., lignin, tannins, or cashew nut shell liquid (CNSL).^19–22^ While these systems have shown some promise and progress, studies exploring vastly different adhesive systems beyond combining natural phenolic resources with phenol and formaldehyde remain limited. Carbohydrates, as an abundant natural resource, are particularly attractive candidates for industrial-scale adhesive production. While the performance of adhesives solely made from native carbohydrates faces challenges (i.e., inferior moisture resistance, strength, and reactivity), adhesives made with modified carbohydrates or their derivatives, along with the addition of synthetic or natural cross-linking agents, offer promising solutions to these challenges.^23–25^

One extensively studied carbohydrate derivative is furfuryl alcohol (FA). FA is produced on an industrial scale from hemicellulose-rich agricultural residues (e.g., bagasse, corn cobs, oat hulls, etc.). Hemicelluloses are decomposed by acid hydrolysis to pentoses and then dehydrated to form furfural, which upon further reduction yields FA. Under acidic conditions, FA is capable of homopolycondensation, first generating linear chains connected via methylene bridges, which subsequently cross-link to form a durable thermoset network (Figure 2).^26–29^

**Figure 2:**
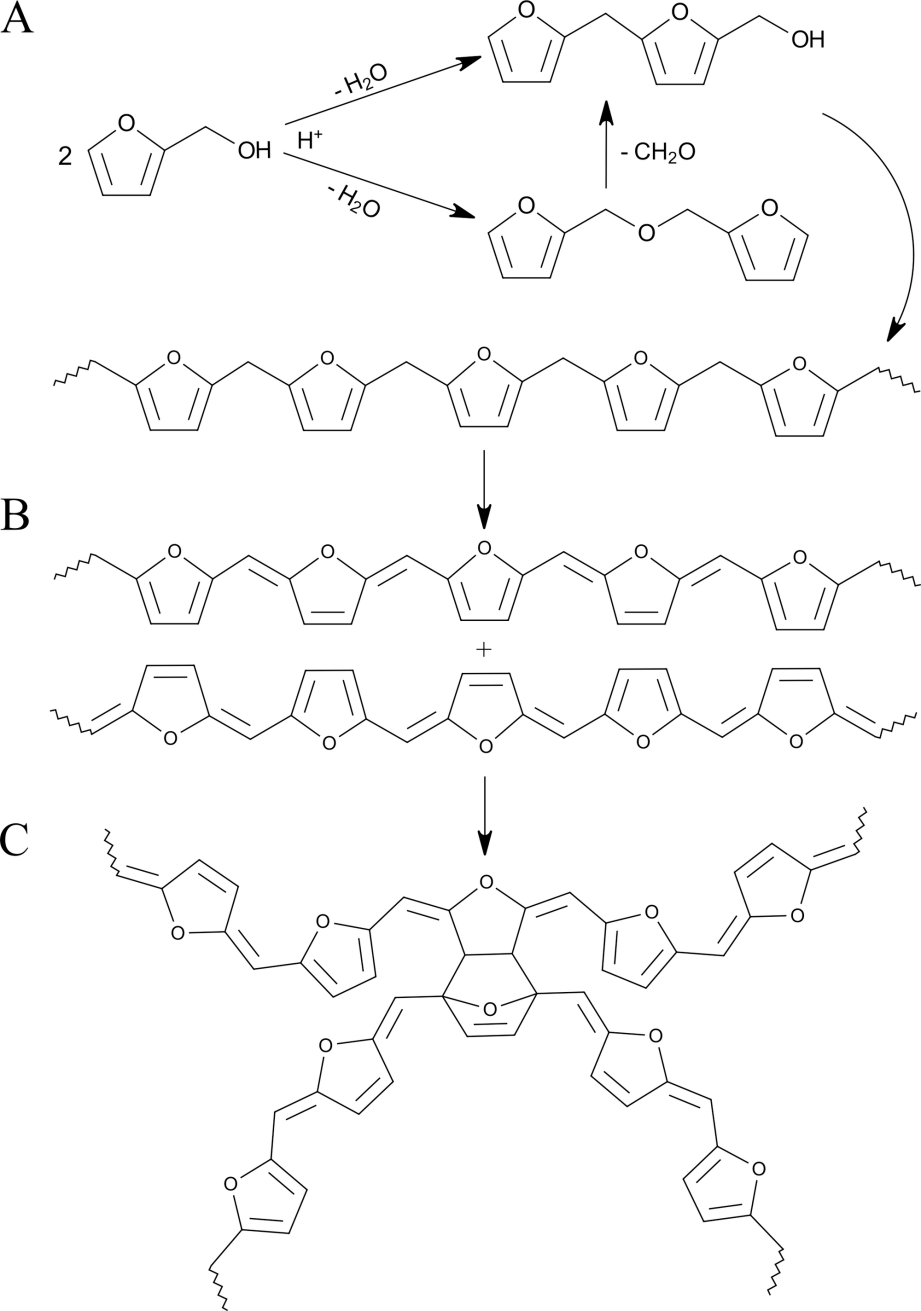
Simplified polymerization mechanism of poly­(furfuryl alcohol): (A) formation of linear chains, (B) formation to the conjugated system, and (C) cross-linking of chains. More detailed graphical data and concepts can be found in Belgacem and Gandini's work.^30^

Resins based on poly­(furfuryl alcohol) (PFA) already find application in the production of foundry cores and molds as well as in the automotive, aerospace, and construction industry.^31–34^ Furthermore, PFA and resins based on FA and coreactants have been extensively studied for the use as wood adhesives and for the production of fiber-reinforced composites.^29,35–39^ PFA also exhibits several properties deemed beneficial for their use in HPL production, such as resistance to corrosive chemicals, fire, and moisture.^26,32^ In addition, the low molecular weight of the monomeric furfuryl alcohol facilitates obtaining low viscosities required for impregnation purposes.

5-Hydroxymethylfurfural (5-HMF) is another carbohydrate derivative that has gained significant scientific attention. Rosenfeld et al. provided a comprehensive review on the use of 5-HMF and its derivatives as a reactive component in adhesive formulations for the wood, glass fiber, and foundry industry.^40^ Generally, 5-HMF is produced through the acid-catalyzed dehydration of hexoses, like glucose and fructose.^41–43^ While there is a small-scale commercial production plant with a capacity of 300 t/a of 5-HMF in operation, the large-scale production is still deemed challenging.^40^ Recently, our group investigated the *in situ* production of a precursor solution rich in 5-HMF targeting its direct application as a reactive compound in adhesive formulations.^41,44^ Further studies explored the combination of this precursor with fructose and amines.^23^ The resulting polymeric adhesive network consists of fructosyl amines, Maillard products, and compounds formed from the reaction of 5-HMF and the amine. Furthermore, humins are formed proposedly from self-condensation of 5-HMF, adding to the multitude of possible reactions and complexity of the system (Figure 3).^44^ A full elucidation of the adhesive system remains incomplete, largely due to the given the complexity of the underlying reactions.^45^

**Figure 3:**
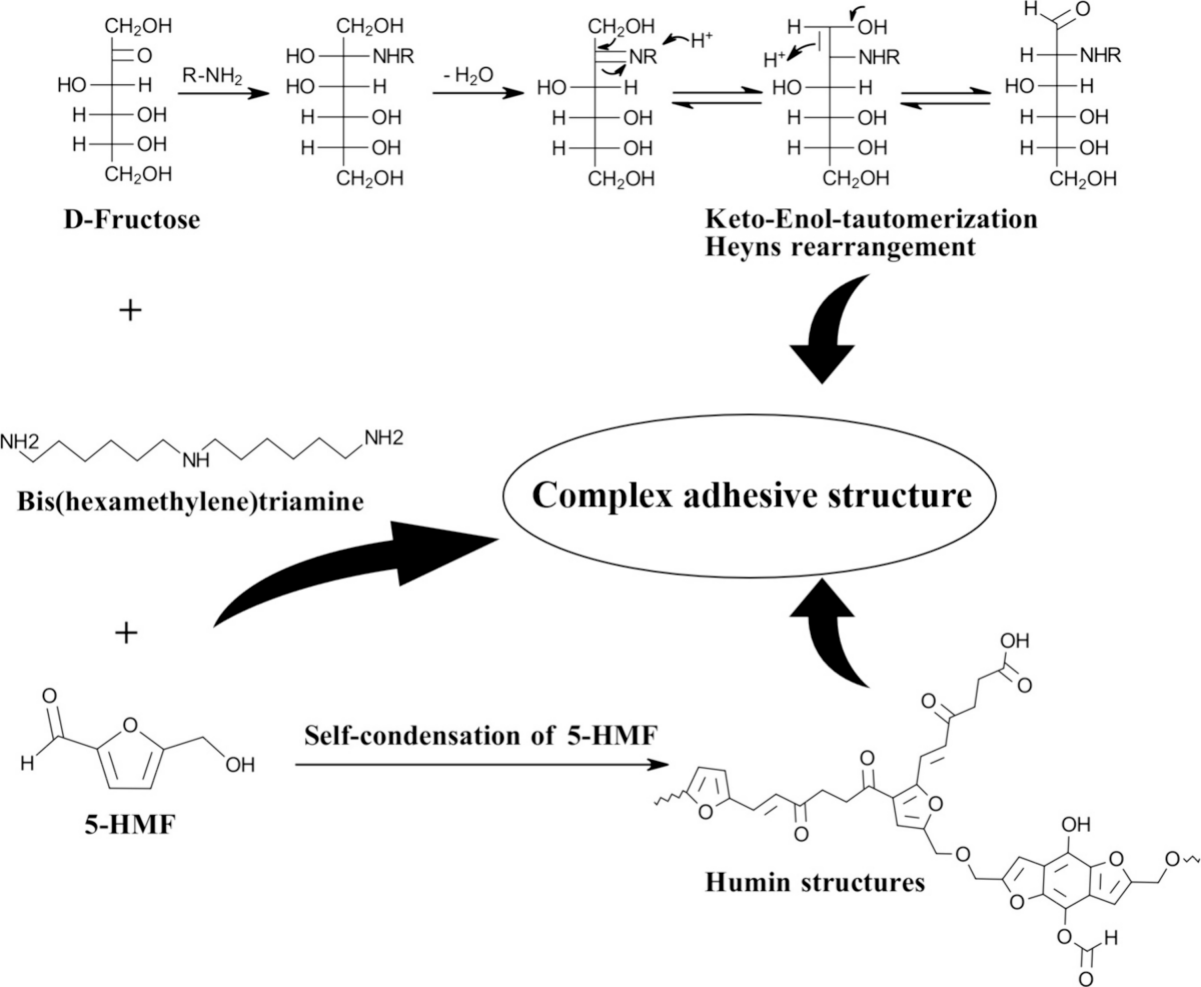
Possible reactions leading to the complex polymeric network of fructose-HMF-amine adhesive. Based on graphical data and concepts from Rosenfeld et al.^45^ and Sailer-Kronlachner et al.^41^ (adapted under the terms of the Creative Commons Attribution 4.0 International License (CC BY 4.0)).

The adhesive system has shown great promise for the production of particleboards and medium-density fiberboards. The findings revealed that the incorporation of 5-HMF reduces the activation energy required for curing, accelerates the development of tensile shear strength, and results in adequate mechanical properties of the final wood-based products.^23,41^ Further characterization of the adhesive system can be found in previous publications by our group.^23,41,44,45^

The industrial requirements that impregnation resins have to fulfill are a challenge for the development of a novel, biobased system. The properties of the final product are highly sensible to the raw material, meaning that changes in the employed kraft paper or resin composition can greatly affect the quality of the boards.^2,46–48^ The suitability of a novel resin for HPL production is largely determined by its interaction with the paper. First, the viscosity of the resin must be low to fully and rapidly penetrate the pores of the paper matrix. Furthermore, the mutual interaction between the surface tension of the resin and the surface free energy of the paper significantly determines their compatibility. Coming from these considerations, kraft papers containing different proportions of recycled fibers were thoroughly characterized and their compatibility to carbohydrate-based impregnation resins synthesized in this work was investigated.

## Experimental Section

### Materials and Chemicals

Three types of commercial kraft papers used for HPL production with varying amounts of recycled fibers (virgin fibers/recycled fibers, 100/0, 85/15, and 0/100) were analyzed. The samples are referred to as VI virgin, MI mixed, and RE recycled paper. Two commercial low-viscous PF impregnation resins (PF, 1:63% solids; PF, 2:60% solids) served as a reference. Due to confidentiality agreements, the supplier of these materials cannot be disclosed.

Fructose syrup FF95 (70.5% dry solids, 95% purity) was supplied by Cargill Deutschland GmbH (Krefeld, Germany). Bis­(hexamethylene)­triamine (BHT) (crystalline, high purity), sodium dithionite (85% purity), furfuryl alcohol (≥98.0% purity), maleic anhydride (≥99.5% purity), and acetone (≥99.8% purity) were supplied by Merck Chemicals Co. (Darmstadt, Germany). Sulfuric acid (96%) was purchased from Carl Roth GmbH & Co. KG (Karlsruhe, Germany).

### Microscopic Analysis

High-vacuum backscattered electron imaging was performed using an Apero VS scanning electron microscope (SEM) (Thermo Scientific, The Netherlands). Samples were attached to the holder using a conductive carbon tape (Agar Scientific Ltd., Essex, UK). Samples were sputter-coated with a 5 nm gold layer under an argon atmosphere using a Leica EM QSG100 coater (Leica Microsystems, Wetzlar, Germany). SEM images were taken at an accelerating voltage of 3 kV with varying magnifications.

Micrographs were taken using a Zeiss Axioplan 2 imaging microscope at 10× magnification.

### Chemical Characterization of Kraft Paper

Acid methanolysis was performed following the protocol described by Sundberg et al.^49^ The extractives content was determined according to TAPPI standard T 204 (cm-97) by subjecting the kraft paper samples to Soxhlet extraction with acetone for 4 h, followed by gravimetric quantification after evaporation. The kappa number of the kraft paper was determined in accordance with TAPPI standard T 236 (om-99). All measurements were performed in triplicate.

### Determination of Surface Free Energy of Kraft Papers

The paper samples were climatized for 48 h at 23 °C and 50% relative humidity before contact angle measurement. The contact angle (θ) of water (2 μL), formamide (2 μL), and diiodomethane (1 μL) on the paper surfaces was measured with a drop shape analyzer DSA 30 (Krüss, Hamburg, Germany) using the sessile drop method (*n* = 10 per reference fluid).^50^ The contact angles of reference fluids were used without compensating for roughness to reflect the true state of paper surfaces, as encountered in industrial practice allowing a relative comparison between the different paper grades. The surface tension (γ_L_) and its polar (γ_L_^p^) and disperse (γ_L_^d^) fractions of the used fluids are provided in the Supporting Information. The surface free energy (γ_S_) was calculated based on the contact angle that was established when the drop stabilized on the paper surface. The polar (γ_S_^p^) and disperse (γ_S_^d^) components of the surface free energy were calculated based on the mean contact angle of the reference fluids (Supporting Information) using the Owens–Wendt–Rable–Kaelble (OWRK) model.^50–53^

### Synthesis of Resins

Two resins from different renewable resources were synthesized, chosen for their good market availability and their high biobased content.

#### Poly­(furfuryl alcohol) Resin

Poly­(furfuryl alcohol) (PFA) was produced by the homopolymerization of FA. FA was dosed into a three-neck flask equipped with a magnetic stirrer, a condenser, and a temperature sensor. Maleic anhydride (2.5 wt %) was added as a catalyst. The reaction mixture was heated to 70 °C and maintained at this temperature for approximately 1.5 h. The solid content of the final adhesive was at 59%.

#### Fructose-HMF-amine Resin

A fructose-HMF-amine (FHA) adhesive was prepared according to Sailer-Kronlachner et al.^23,41^ Briefly, a precursor solution rich in 5-hydroxymethylfurfural (5-HMF) was obtained by dehydrating fructose syrup, using sulfuric acid as a catalyst. The precursor, along with additional fructose syrup, was heated to 40 °C in a three-neck flask. Bis­(hexamethylene)­triamine (BHT) was added, and the reaction mixture was further heated to 60 °C for 30 min. The resulting adhesive was diluted with 5 wt % water, attaining a solid content of 57%.

### Viscosity Measurements of Resins

Viscosity measurements were performed using an MCR 302 rheometer (Anton Paar GmbH) at a shear rate of 100 1/s and at a temperature of 23 °C (*n* = 10 per resin). A cone plate with a cone angle of 1°, a diameter of 50.0 mm, and a gap size of 1 mm was used.

### Surface Tension Measurements of Resins

The resins' surface tensions were measured with a drop shape analyzer 30 (Krüss, Hamburg, Germany) using the pendant drop method (*n* = 10 per resin). The polar and disperse components were determined through contact angle measurements of the adhesives on a microscope glass slide coated with paraffin wax (*n* = 10 per resin).

### Contact Angle Measurements of Resins on Kraft Paper

The time-dependent decrease in resin contact angles on the paper surfaces was measured to evaluate their wetting kinetics and penetration behavior on the paper. Contact angle measurements (2 μL) were conducted with a drop shape analyzer 30 by Krüss (Hamburg, Germany) using the sessile drop method (*n* = 5 per resin). Contact angles of the resins were not accounted for the roughness of the paper to reflect their practical application conditions during the impregnation process.

### Impregnation of Kraft Paper with Resins

Paper samples (30 × 30 mm) were climatized for 48 h at standard climate conditions of 23 °C and 50% relative humidity. The samples were then placed into a Petri dish containing the resins, and the time required for the resins to saturate the material was measured (*n* = 5 per resin). This method was included to allow comparison of contact angle measurements with standard evaluation practices applied in the industry for resin-paper compatibility. All examined samples were of comparable thickness and grammage (Supporting Information, Table S4), ensuring a consistent basis for comparison.

## Results and Discussion

A thorough characterization of the paper properties was performed to explain wettability differences before assessing resin compatibility.

### Characterization of Kraft Papers

Microscopy demonstrated considerable contamination in MI and especially RE samples. This was evident from the presence of small, multicolored plastic residues scattered across the paper surface (Supporting Information). These fragments are caused by the use of a diverse mixture of postconsumer waste paper, which often contains plastic contaminants such as stickers or tags.^54^ SEM images also revealed differences in the structural morphology of the paper grades (Figure 4 and the Supporting Information). While VI and MI samples exhibited only minor differences in SEM, RE samples with higher recycled fiber content showed increasingly fragmented and frayed fibers due to deterioration from recycling. Additionally, two-sidedness of the paper samples was observed, a result of the papermaking process using Fourdrinier machines. Coarser fibers settle first to form the rougher wire (bottom) side, while finer fibers and fillers accumulate on the felt (top) side, creating a smoother, denser surface.^55–57^ SEM images demonstrated a more open fiber network on the wire side. In addition, samples containing recycled fibers showed a more pronounced accumulation of fines and inorganic matter, particularly in RE.

**Figure 4:**
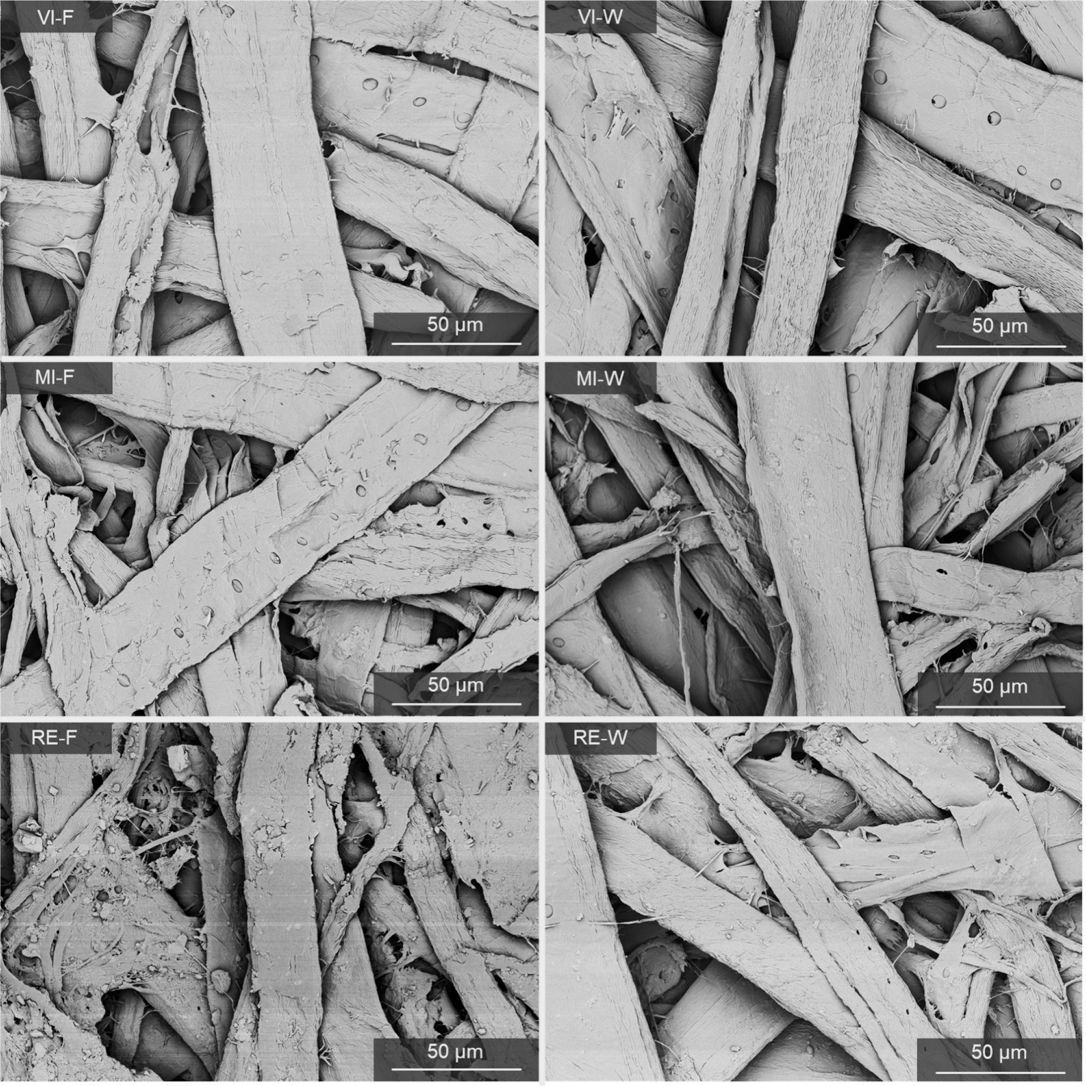
SEM images at 2000× magnification of kraft papers' felt (F) and wire (W) side: VI-F (left), VI-W (right), MI-F (left), MI-W (right), RE-F (left), and RE-W (right).

The surface free energy of a material is strongly influenced by its chemical composition. Paper wettability is mainly affected by noncellulosic constituents, particularly by the content of lignin and extractives.^58–61^ This in turn impacts the bonding performance with impregnation resins in the final HPL product.^1^ Therefore, the paper samples were further analyzed for their chemical composition.

Acid methanolysis was performed to identify the wood species used. While the carbohydrate content of RE and MI was similar, they deviated by around 10.6% from VI, reflecting the degradation and loss of wood polysaccharides during the recycling process (Figure 5 and the Supporting Information). RE showed higher levels of xylose, arabinose, and 4-*O*-methyl-d-glucuronic acid (MeGlcA), suggesting hardwood origin. In contrast, VI and MI displayed elevated levels of galactose and mannose, characteristic of softwood hemicelluloses.^62,63^ The hemicellulose profile of MI suggests a mixed fiber composition of softwood and hardwood, whereas VI shows characteristics of softwood-derived fibers, with high mannose and galactose but lacking amounts of MeGlcA.

**Figure 5:**
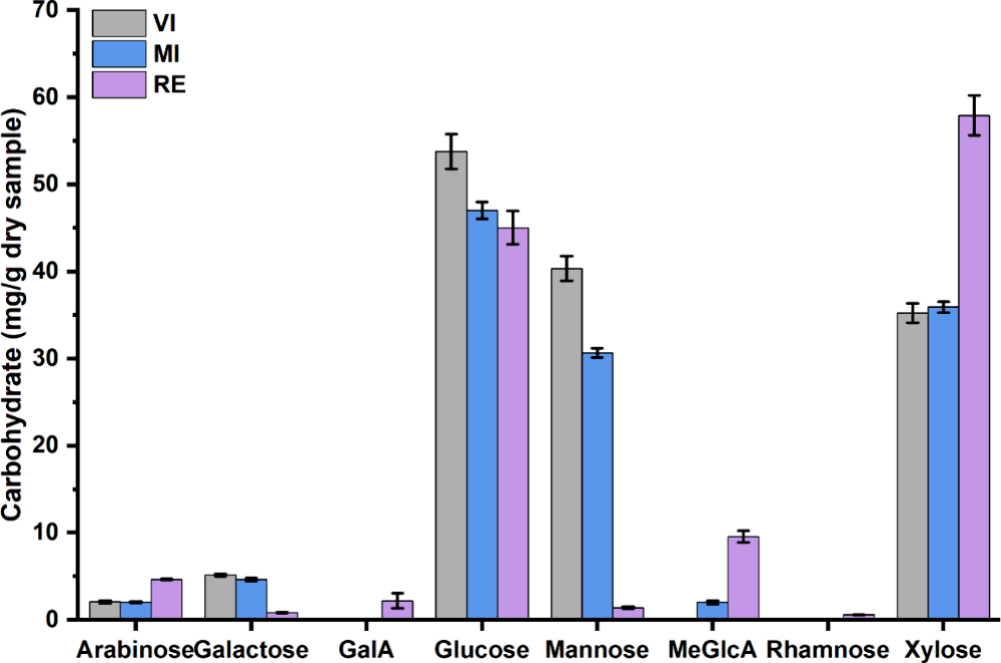
Hemicellulose content and monosaccharide composition (arabinose, galactose, galacturonic acid (GalA), glucose, mannose, 4-*O*-methyl-d-glucuronic acid (MeGlcA), rhamnose, and xylose) of kraft paper samples determined by acidic methanolysis followed by GC-MS analysis.

The kappa number was used as an estimate for the residual lignin content (Table 1). The kappa number of VI was around 30, slightly higher for MI and nearly twice as high for RE. The recycled paper studied here is made from a mixture of postconsumer waste paper, with a significant portion derived from the reuse of old corrugated cardboard (OCC). OCC has been reported to have high kappa numbers even exceeding 100, which explains the elevated values in the recycled paper studied here.^64–66^

**Table 1:** Chemical Composition of Kraft Paper Samples

paper type	kappa number	extractives content (%)	ash (%) (data from the manufacturer)
VI	30.2 (±0.31)	0.09 (±0.03)	<1
MI	33.9 (±0.07)	0.14 (±0.03)	2.8
RE	58.6 (±0.93)	1.23 (±0.02)	7.5

RE showed higher levels of extractives than VI and MI, suggesting that the recycling process contributes to an accumulation of acetone-soluble substances. Residual printing inks, pigments, adhesives, or other contaminants from the paper’s previous use may result in additional extractives not present in virgin pulp samples.

The ash content reported from the paper supplier varied among paper grades, indicating differences in inorganic filler use. Fillers, like calcium carbonate or kaolin, are commonly added to improve optical properties as well as for cost reduction of the process.^54,67–71^

### Surface Free Energy of Kraft Paper

VI showed the highest surface free energy for both polar and disperse components, while MI and RE showed slightly lower values. While both sides of the paper showed relatively similar values for VI and MI, the reduction in surface free energy was more pronounced on the felt side of RE (Figure 6).

**Figure 6:**
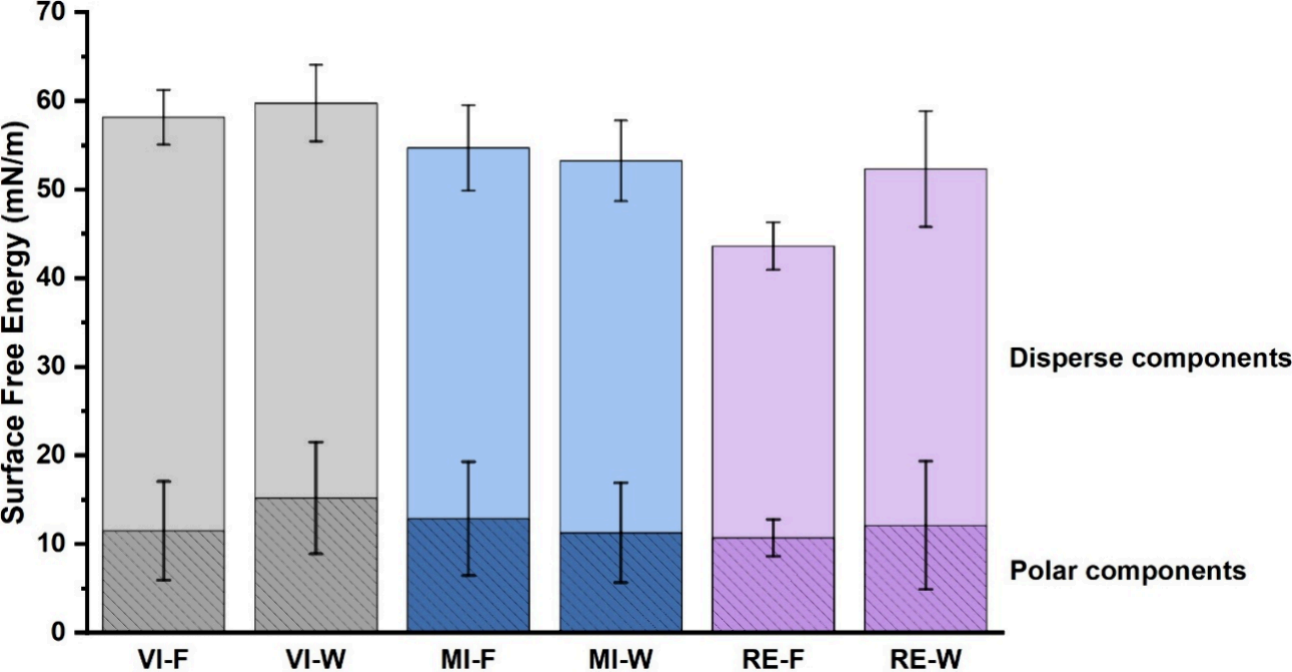
Surface free energy of kraft papers' felt (F) and wire (W) side.

The reduced surface free energy of MI and RE can be attributed to changes induced by the chemical and mechanical treatments that the fibers underwent during recycling. Recycled paper typically has a lower surface free energy than virgin paper due to fiber modification during processing. Cellulose fibrils are not fully rehydrated when resuspended in water. Subsequent drying causes the cell walls to collapse, forming intramolecular hydrogen bonds that are not fully cleaved upon rehydration, rendering the paper more hydrophobic.^72–74^ The discussed differences in chemical composition, i.e., higher levels of residual lignin and extractives, also contribute to the reduction in surface free energy. Bäckström et al. investigated the influence of the residual lignin and extractives present in paper on their surface energy.^58^ Samples with a higher kappa number and extractives content were found to have lower surface free energy values.^58^ Moreover, the presence of additives, inorganic fillers, and impurities can have a substantial effect on the wettability of paper.^1,2^ The accumulation of inorganic fillers is likely responsible for the greater decrease in surface free energy of the felt side of RE.

### Viscosity and Surface Tension of Resins

The viscosity of impregnation resins typically ranges from 15 to 100 mPa s and should not exceed 300 mPa s to ensure proper resin flow into the paper matrix.^2,48^ The viscosities of all examined resins were sufficiently low for impregnation purposes. PF1 and PF2 showed similar values of 74 and 77 mPa s, while the biobased resins had higher viscosities little above 100 mPa s (Figure 7).

**Figure 7:**
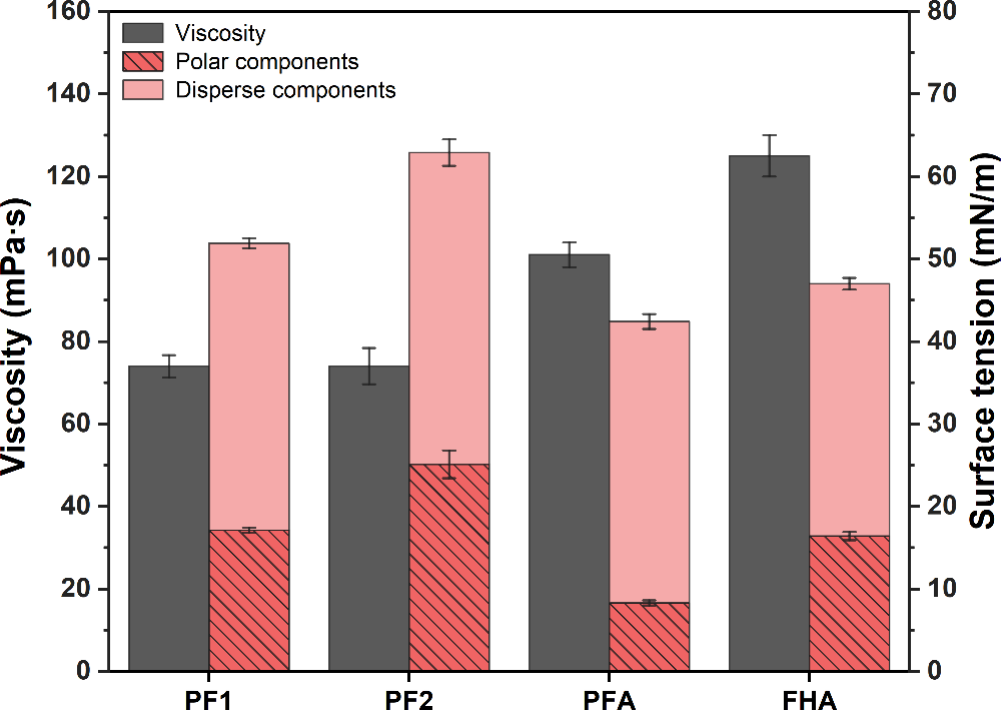
Surface tension composition and viscosity of resins (*n* = 10).

Apart from viscosity, the surface tension of impregnation resins is a crucial factor influencing the saturation of the paper substrate and thus influencing the final product. High values for both properties can lead to poor resin flow and uneven resin distribution, resulting in defects in the final HPL board.^2^ Generally, the surface tension of the liquid resin must be lower than the surface free energy of the solid material to achieve adequate wetting. Their polar and dispersive components should be similar to enhance the compatibility of both substrates.^75,76^ Significant differences were observed in the surface tension values. PF1 had a surface tension of around 52 mN/m, while PF2 had the highest surface tension of 63 mN/m with a notably higher polar fraction. This difference may be attributed to variations in the synthesis conditions. Thébault et al. found that PF resins exhibit higher surface tensions and polarity when synthesized at lower pH values and for shorter durations.^48^ In contrast, the biobased resins had lower surface tensions, with PFA attaining the lowest value of around 42 mN/m and a reduced polar fraction. FHA attained a surface tension of 47 mN/m, with a lower disperse fraction and a polar fraction comparable to PF1.

### Contact Angle Measurements of Resins on Kraft Paper

Contact angle measurements of the resins on the kraft paper samples further highlighted the differences among the materials (Figure 8). VI exhibited the highest wettability with all resins, while the wettability gradually decreased as the recycled fiber content increased. Additionally, the wire side of all samples showed superior wettability than the felt side. This trend aligns well with their determined chemical composition, structural morphology, and surface free energy.

**Figure 8:**
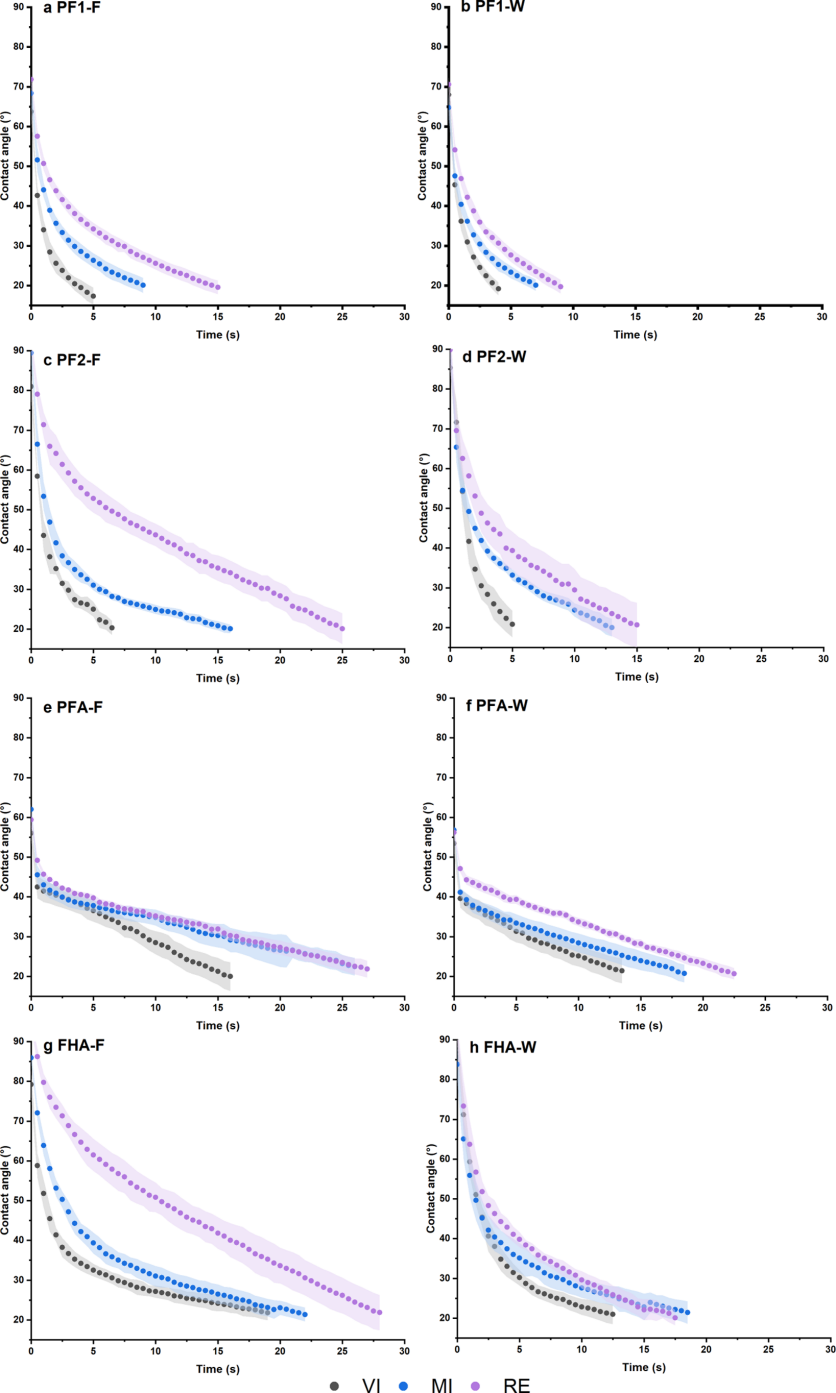
Mean contact angle decrease (*n* = 5) of resins on kraft papers' felt (F) and wire (W) side.

While the difference in decreasing contact angles between PF1 and PF2 was less pronounced for VI, large variations were observed for MI and RE. Compared to PF1, PF2 with higher polarity took longer to reach the same contact angle on these surfaces, especially on the felt side of RE. PFA had a lower initial contact angle, likely due to its significantly lower polarity, while its penetration into the different papers was slower and more gradual. FHA demonstrated a shorter to comparable penetration rate into the wire side compared to PFA, and the slowest penetration rate of all resins on the felt sides of the papers.

Despite the differences in the rate of contact angle decrease, the biobased resins still showed a similar decline compared to PF2 across all paper types. From a wettability point of view, the longer penetration time of the biobased resins synthesized here may thus still be feasible for impregnation purposes, considering that both PF resins and all kraft papers investigated here are currently in industrial and commercial use for paper impregnation and HPL production.

### Impregnation of Kraft Paper with Resins

While contact angle measurements provide quantifiable data on resin-paper interactions, they do not accurately reflect the impregnation process. In the industry, it is common practice to gain initial information on the impregnability of a resin by immersing a paper sample in a resin bath and measuring the time required for the resin to migrate from the side in contact with the resin to the upper surface of the paper. Although this technique is not standardized, it may capture the dynamic behavior of resin imbibition more effectively under conditions that more closely resemble the impregnation process. As such, it provides a practical evaluation of resin performance, serving as a useful and rapid complement to our contact angle analysis. All of the paper samples were of comparable thickness and grammage, with only negligible variations (Supporting Information, Table S4). This enabled direct comparison of resin penetration, unaffected by potential variations in the dimensions of the samples.

The trend of wettability for the different papers' felt and wire sides was consistent with their determined surface free energy. PFA exhibited the fastest imbibition rate, followed by PF1, FHA, and PF2 (Figure 9). While these observations align well with the determined surface tensions of the individual resins, they deviate from the determined speed of contact angle decrease. These differences likely arise from the localized nature of the wetting process during contact angle measurements, where the resin interacts with only a limited area of the paper. In contrast, during the immersion test, the resin saturates the paper from all exposed directions, allowing for a faster and complete wetting of the porous substrate with the resin. Thus, the discrepancy between the slower penetration observed for the biobased resins in the contact angle analysis and their faster imbibition rate during impregnation might arise from the differences in the total wetted area of the sample. This underscores the importance of using both contact angle measurements and saturation tests. Contact angle analysis provides measurable data on the wetting behavior, while saturation time measurements reflect the resin flow and absorption conditions closer to HPL production.

**Figure 9:**
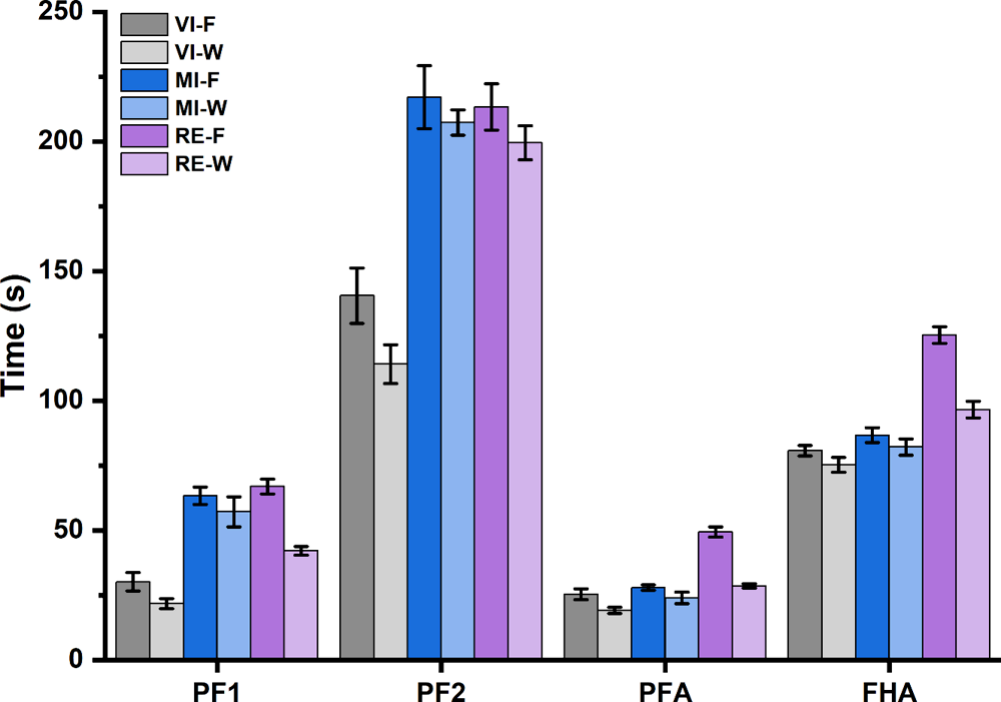
Time until full saturation of paper samples with individual impregnation resins (*n* = 5).

## Conclusions

This study focused on the characterization of recycled paper and biobased impregnation resins for potential HPL production. Different grades of kraft paper, with varying recycled fiber content, were analyzed for their chemical composition, structural morphology, and resulting wettability. Recycled paper exhibited a lower surface free energy and wettability compared to paper made with virgin fibers. Apart from factors like modifications stemming from fiber recycling, this can be explained by a higher lignin, extractives, and inorganic filler content. Additionally, recycled paper showed increased two-sidedness, with cellulose fines and inorganic matter more concentrated on the felt side. Biobased resins were synthesized from carbohydrates and/or their derivatives and characterized with regard to wetting behavior. Contact angle measurements and impregnation trials revealed vast differences in the penetration behavior between the PF resins as well as the biobased resins. Despite this, the biobased resins still showed comparable performance to the commercial PF impregnation resin with higher polarity. While the examined biobased resins and recycled paper currently face higher production costs compared to traditional materials, they offer significant potential due to the growing demand for sustainable solutions. Advancements in research could help make these materials more competitive in the future. Therefore, further work will focus on optimizing synthesis conditions and investigating drying and pressing parameters to enhance their performance, with the aim of making them competitive with conventional PF-based HPL.

## Supplementary Material



## Notes

Surface tensions of reference fluids for SFE determination; surface free energy of paraffin wax; mean contact angles of reference liquids on the kraft paper surface; physical properties of paper samples; SEM images at 250× magnification of kraft papers; microscopy images at 10× magnification of kraft papers; carbohydrate composition of kraft paper samples determined by acidic methanolysis and GC-MS analysis (PDF)

## References

[1] Figueiredo A. B., Evtuguin D. V., Monteiro J., Cardoso E. F., Mena P. C., Cruz P. (2011). Structure–Surface Property Relationships of Kraft Papers:
Implication on Impregnation with Phenol–Formaldehyde Resin. Ind. Eng. Chem. Res..

[2] Thébault M., Kandelbauer A., Müller U., Zikulnig-Rusch E., Lammer H. (2017). Factors Influencing
the Processing and Technological
Properties of Laminates Based on Phenolic Resin Impregnated Papers. Eur. J. Wood Prod..

[3] Burdurlu E., Ozgenc O. (2009). Effect of Different
Layer Structures on Some Resistance
Characteristics of High Pressure Laminates. For. Prod. J..

[4] Henriques, A. ; Coelho, C. ; Ferra, J. M. ; Martins, J. M. ; Magalhães, F. D. ; Carvalho, L. 1. Introduction of Advanced Functionalities in Laminates for Wood-Based Panels: Surface Quality Evaluation. In Wood Composites; Davim, J. P. ; Aguilera, A. , Eds.; De Gruyter, 2017; pp 1–32. 10.1515/9783110416084-001.

[5] Magina S., Santos M., Ferra J., Cruz P., Portugal I., Evtuguin D. (2016). High Pressure Laminates with Antimicrobial
Properties. Materials.

[6] Martins J. M., Almeida M. L., Coelho C. M., Ferra J., Carvalho L. H. (2015). A New Methodology
to Evaluate the Cure of Resin-Impregnated Paper for HPL. J. Adhes..

[7] Ji Y., Xia Q., Cui J., Zhu M., Ma Y., Wang Y., Gan L., Han S. (2021). High Pressure
Laminates Reinforced with Electrospun
Cellulose Acetate Nanofibers. Carbohydr. Polym..

[8] Bayatkashkoli A., Ramazani O., Keyani S., Mansouri H. R., Madahi N. K. (2018). Investigation
on the Production Possibilities of High Pressure Laminate from Borax
and Recycled Papers as a Cleaner Product. J.
Clean. Prod..

[9] Alkan Ü. B., Kızılcan N., Bengü B. (2025). Lignosulfonate
and Glycidyl Ether Modified Urea Formaldehyde Wood Adhesives for Interior
Particleboard Production. PRT.

[10] Oktay S., Pizzi A., Köken N., Bengü B. (2024). Tannin-Based
Wood Panel Adhesives. Int. J. Adhes. Adhes..

[11] Oktay S., Kızılcan N., Bengü B. (2024). Environment-Friendly Cornstarch and
Tannin-Based Wood Adhesives for Interior Particleboard Production
as an Alternative to Formaldehyde-Based Wood Adhesives. PRT.

[12] Oktay S., Kızılcan N., Bengü B. (2021). Development
of Bio-Based Cornstarch
- Mimosa Tannin - Sugar Adhesive for Interior Particleboard Production. Ind. Crop. Prod..

[13] Kizilcan N. (2012). Lignosulphonate
Modified Ketonic Resins. PRT.

[14] Alkan Ü. B., Kızılcan N., Bengü B. (2022). Urea Glyoxal
and Urea Melamine Glyoxal Wood Adhesives Hardened with Acid Ionic
Liquid for Particleboard Pressing. Eur. J. Wood
Prod..

[15] Oktay S., Pizzi A., Köken N., Bengü B. (2024). Chemical Modification
Techniques of Corn Starch for Synthesis Wood Adhesive. Int. J. Adhes. Adhes..

[16] Bliem P., Zinovyev G., Van Herwijnen H. W. G. (2025). High-Performance
Lignin-Phenol-Formaldehyde
Resins Synthesised with Hardwood Lignin from Continuous Batch Cooking-CBC
Process. J. Adhes..

[17] Averina E., Konnerth J., Van Herwijnen H. W. G. (2023). Protein-Based
Glyoxal–Polyethyleneimine-Crosslinked
Adhesives for Wood Bonding. J. Adhes..

[18] Zhang Q., Lei H., Du G., Pizzi A., Song J., Cao L., Puangsin B., Xi X. (2024). Easy Preparation, High Water Resistance
Glucose-Based Environment-Friendly Wood Adhesives. ACS Sustainable Chem. Eng..

[19] Thébault M., Kutuzova L., Jury S., Eicher I., Zikulnig-Ruschand E.
M., Kandelbauer r. (2020). Effect of
Phenolation, Lignin-Type and Degree of Substitution
on the Properties of Lignin-Modified Phenol-Formaldehyde Impregnation
Resins: Molecular Weight Distribution, Wetting Behavior, Rheological
Properties and Thermal Curing Profiles. J. Renewable
Mater..

[20] Thébault M., Müller U., Kandelbauer A., Zikulnig-Rusch E., Lammer H. (2017). Review on Impregnation
Issues in Laminates Manufacture:
Opportunities and Risks of Phenol Substitution by Lignins or Other
Natural Phenols in Resins. Eur. J. Wood Prod..

[21] Ghorbani M., Mahendran A. R., van Herwijnen H. W. G., Liebner F., Konnerth J. (2018). Paper-Based
Laminates Produced with Kraft Lignin-Rich Phenol-Formaldehyde Resoles
Meet Requirements for Outdoor Usage. Eur. J.
Wood Prod..

[22] Ghorbani M., Liebner F., Van Herwijnen H. W. G., Pfungen L., Krahofer M., Budjav E., Konnerth J. (2016). Lignin Phenol
Formaldehyde Resoles:
The Impact of Lignin Type on Adhesive Properties. BioResources.

[23] Thoma C., Solt-Rindler P., Sailer-Kronlachner W., Rosenau T., Potthast A., Konnerth J., Pellis A., van Herwijnen H. W. G. (2021). Carbohydrate-Hydroxymethylfurfural-Amine
Adhesives: Chemorheological Analysis and Rheokinetic Study. Polymer.

[24] Patel A. K., Mathias J.-D., Michaud P. (2013). Polysaccharides as
Adhesives. Rev. Adhes. Adhesives.

[25] Dunky, M. Wood Adhesives Based on Natural Resources: A Critical Review: Part II. Carbohydrate-Based Adhesives. In Progress in Adhesion and Adhesives; Mittal, K. L. , Ed.; Wiley, 2021; pp 337–382. 10.1002/9781119846703.ch9.

[26] Gandini A. (1997). Furans in
Polymer Chemistry. Prog. Polym. Sci..

[27] Sadler J. M., Yeh I., Toulan F. R., McAninch I. M., Rinderspacher B. C., La Scala J. J. (2018). Kinetics Studies and Characterization of Poly­(Furfuryl
Alcohol) for Use as Bio-based Furan Novolacs. J. Appl. Polym. Sci..

[28] Montero A. L., Montero L. A., Martínez R., Spange S. (2006). Ab Initio Modelling
of Crosslinking in Polymers. A Case of Chains with Furan Rings. J. Mol. Struct. (THEOCHEM).

[29] Belgacem, M. N. ; Gandini, A. Handbook of Adhesive Technology. In Handbook of adhesive technology; Pizzi, A. ; Mittal, K. L. , Eds.; Marcel Dekker: New York (N. Y.), 2003.

[30] Belgacem, M. N. ; Gandini, A. Chapter 6 - Furan Derivatives and Furan Chemistry at the Service of Macromolecular Materials. In Monomers, polymers and composites from renewable resources; Elsevier: Amsterdam; Boston, 2008.

[31] Choura M., Belgacem N. M., Gandini A. (1996). Acid-Catalyzed Polycondensation of
Furfuryl Alcohol: Mechanisms of Chromophore Formation and Cross-Linking. Macromolecules.

[32] Iroegbu A. O., Hlangothi S. P. (2019). Furfuryl
Alcohol a Versatile. Eco-Sustainable Compound
in Perspective. Chem. Afr..

[33] Iroegbu A. O., Sadiku E. R., Ray S. S., Hamam Y. (2020). Sustainable Chemicals:
A Brief Survey of the Furans. Chem. Afr..

[34] Iroegbu A. O. C., Ray S. S. (2024). On the Chemistry of Furfuryl Alcohol Polymerization:
A Review. J. Polym. Sci..

[35] Zhang J., Liu B., Zhou Y., Essawy H., Chen Q., Zhou X., Du G. (2021). Preparation
of a Starch-Based Adhesive Cross-Linked with Furfural,
Furfuryl Alcohol and Epoxy Resin. Int. J. Adhes.
Adhes..

[36] Xi X., Wu Z., Pizzi A., Gerardin C., Lei H., Du G. (2020). Furfuryl Alcohol-Aldehyde
Plywood Adhesive Resins. J. Adhes..

[37] Pin J.-M., Guigo N., Mija A., Vincent L., Sbirrazzuoli N., van der Waal J. C., de Jong E. (2014). Valorization of Biorefinery Side-Stream
Products: Combination of Humins with Polyfurfuryl Alcohol for Composite
Elaboration. ACS Sustainable Chem. Eng..

[38] Sangregorio A., Muralidhara A., Guigo N., Marlair G., De Jong E., Sbirrazzuoli N. (2021). Natural Fibre
Composites with Furanic Thermoset Resins.
Comparison between Polyfurfuryl Alcohol and Humins from Sugar Conversion.
Composites Part C: Open. Access.

[39] Kumar R., Anandjiwala R. D. (2013). Compression-Moulded
Flax Fabric-Reinforced Polyfurfuryl
Alcohol Bio-Composites: Mechanical and Thermal Properties. J. Therm. Anal. Calorim..

[40] Rosenfeld C., Konnerth J., Sailer-Kronlachner W., Rosenau T., Potthast A., Solt P., Van Herwijnen H. W. G. (2020). Hydroxymethylfurfural
and Its Derivatives:
Potential Key Reactants in Adhesives. ChemSusChem.

[41] Sailer-Kronlachner W., Thoma C., Böhmdorfer S., Bacher M., Konnerth J., Rosenau T., Potthast A., Solt P., van Herwijnen H. W. G. (2021). Sulfuric
Acid-Catalyzed Dehydratization of Carbohydrates for the Production
of Adhesive Precursors. ACS Omega.

[42] Van
Putten R.-J., Van Der Waal J. C., De Jong E., Rasrendra C. B., Heeres H. J., De Vries J. G. (2013). Hydroxymethylfurfural, A Versatile
Platform Chemical Made from Renewable Resources. Chem. Rev..

[43] Solt P., Konnerth J., Gindl-Altmutter W., Kantner W., Moser J., Mitter R., van Herwijnen H. W. G. (2019). Technological Performance of Formaldehyde-Free
Adhesive Alternatives for Particleboard Industry. Int. J. Adhes. Adhes..

[44] Sailer-Kronlachner W., Rosenfeld C., Böhmdorfer S., Bacher M., Konnerth J., Rosenau T., Potthast A., Geyer A., van Herwijnen H. W. G. (2024). Scale-Up
of Production of 5-Hydroxymethylfurfural-Rich Adhesive Precursors
and Structural Features of Humin Side Products. Biomass Conv. Bioref..

[45] Rosenfeld C., Sailer-Kronlachner W., Konnerth J., Sol-Rindler P., Pellis A., Rosenau T., Potthast A., Van Herwijnen H. W. G. (2022). Hydroxymethylfurfural:
A Key to Increased Reactivity and Performance of Fructose-Based Adhesives
for Particle Boards. Ind. Crop. Prod..

[46] Clark, J. d’A. Pulp Technology and Treatment for Paper, 2nd ed., rev.enl.; A Pulp & paper book; M. Freeman Publications: San Francisco, 1985.

[47] Stock Preparation and Wet End. In Papermaking; Gullichsen, J. ; Paulapuro, H. , Eds.; Papermaking science and technology; Fapet Oy; TAPPI Press: Helsinki, Finland; Atlanta, GA, 2000.

[48] Thébault M., Kandelbauer A., Zikulnig-Rusch E., Putz R., Jury S., Eicher I. (2018). Impact of
Phenolic Resin Preparation on Its Properties
and Its Penetration Behavior in Kraft Paper. Eur. Polym. J..

[49] Sundberg A., Sundberg K., Lillandt C., Holmhom B. (1996). Determination of Hemicelluloses
and Pectins in Wood and Pulp Fibres by Acid Methanolysis and Gas Chromatography. Nord. Pulp Pap. Res. J..

[50] DIN 55660–2:2011–12, Paints and Varnishes - Wettability - Part 2: Determination of the Free Surface Energy of Solid Surfaces by Measuring the Contact Angle, 2011.

[51] Kaelble D.
H. (1970). Dispersion-Polar
Surface Tension Properties of Organic Solids. J. Adhes..

[52] Owens D. K., Wendt R. C. (1969). Estimation of the Surface Free Energy
of Polymers. J. Appl. Polym. Sci..

[53] Rabel W. (1971). Einige Aspekte
Der Benetzungstheorie Und Ihre Anwendung Auf Die Untersuchung Und
Veränderung Der Oberflächeneigenschaften von Polymeren. Farbe Lack.

[54] Pulp and Paper Chemistry and Technology: Vol. 2: Pulping Chemistry and Technology; Gellerstedt, G. ; Henriksson, G. ; Ek, M. , Eds.; Walter de Gruyter: Berlin, 2009.

[55] Hernandez, R. J. ; Selke, S. E. ; Lawal, S. A. Packaging: Papers for Sacks and Bags. In Reference Module in Materials Science and Materials Engineering; Elsevier, 2018; p B9780128035818112238. 10.1016/B978-0-12-803581-8.11223-8.

[56] Handbook of Paper and Board, 1st ed.; Holik, H. , Ed.; Wiley, 2006. 10.1002/3527608257.

[57] Sahin H. T., Manolache S., Young R. A., Denes F. (2002). Surface Fluorination
of Paper in CF4-RF Plasma Environments. Cellulose.

[58] Bäckström M., Fellers C., Htun M. (1999). The Influence of Kappa Number and
Surface Energy on Paper-to-Paper Friction. Nord.
Pulp Pap. Res. J..

[59] Chaiarrekij S., Apirakchaiskul A., Suvarnakich K., Kiatkamjornwong S. (2011). Kapok I: Characteristics
of Kapok Fibre as a Potential Pulp Source for Papermaking. BioResources.

[60] Shen W., Parker I. H., Sheng Y. J. (1998). The Effects
of Surface Extractives
and Lignin on the Surface Energy of Eucalypt Kraft Pulp Fibres. J. Adhes. Sci. Technol..

[61] Dong L., Hu H., Cheng F., Yang S. (2015). The Water Resistance of Corrugated
Paper Improved by Lipophilic Extractives and Lignin in APMP Effluent. J. Wood Sci..

[62] Shinde R., Shahi D. K., Mahapatra P., Naik S. K., Thombare N., Singh A. K. (2022). Potential of Lignocellulose
Degrading Microorganisms
for Agricultural Residue Decomposition in Soil: A Review. J. Environ. Manag..

[63] Anekwe, I. M. S. ; Khotseng, L. ; Isa, Y. M. The Place of Biofuel in Sustainable Living; Prospects and Challenges. In Comprehensive Renewable Energy; Elsevier, 2022; pp 226–258. 10.1016/B978-0-12-819727-1.00068-6.

[64] Zambrano F., Marquez R., Jameel H., Venditti R., Gonzalez R. (2021). Upcycling
Strategies for Old Corrugated Containerboard to Attain High-Performance
Tissue Paper: A Viable Answer to the Packaging Waste Generation Dilemma. Resour. Conserv. Recycl..

[65] Aguilar-Rivera N. (2021). Emerging Technology
for Sustainable Production of Bleached Pulp from Recovered Cardboard. Clean Technol. Environ. Policy.

[66] Danielewicz D., Surma-Ślusarska B. (2015). Properties
of Bleached Pulps from
Low and High Kappa Number Old Corrugated Containers (OCC). Fibres Text. East. Eur..

[67] Seo J., Lee J. G., Thriveni T., Baek C. S., Ahn J.-W. (2014). Improving
Recycled Fiber by Applying In-Situ Aragonite Calcium Carbonate Formation
Process. Mater. Trans..

[68] Roberts, J. C. Physical and Chemical Aspects of the Use of Fillers in the Paper. In Paper Chemistry; Blackie & Son Ltd.: Glasgow, 1991; pp 162–167.

[69] Liendo F., Arduino M., Deorsola F. A., Bensaid S. (2022). Factors Controlling
and Influencing Polymorphism, Morphology and Size of Calcium Carbonate
Synthesized through the Carbonation Route: A Review. Powder Technol..

[70] Hubbe M. A., Gardner D. J., Shen W. (2015). Contact Angles and
Wettability of
Cellulosic Surfaces: A Review of Proposed Mechanisms and Test Strategies. BioResources.

[71] Bajpai, P. Pulp and Paper Making Process. In Bleach Plant Effluents from the Pulp and Paper Industry; SpringerBriefs in Applied Sciences and Technology; Springer: Heidelberg, 2013; pp 7–11. 10.1007/978-3-319-00545-4_2.

[72] Brancato A., Walsh F. L., Sabo R., Banerjee S. (2007). Effect of Recycling
on the Properties of Paper Surfaces. Ind. Eng.
Chem. Res..

[73] Fernandes
Diniz J. M. B., Gil M. H., Castro J. A. A. M. (2004). Hornification
- Its Origin and Interpretation in Wood Pulps. Wood Sci. Technol..

[74] Tze W. T., Gardner D. J. (2001). Contact Angle and IGC Measurements
for Probing Surface-Chemical
Changes in the Recycling of Wood Pulp Fibers. J. Adhes. Sci. Technol..

[75] Roberts, R. J. Liquid Penetration into Paper. Ph.D. Thesis, Australian National University, Canberra, 2004.

[76] Cosgrove, T. Colloid Science: Principles, Methods and Applications, 1st ed.; Wiley, 2005. 10.1002/9781444305395.

