# Supplementary material for: Wettability of Kraft Paper with Biobased Impregnation Resins for the Production of High-Pressure Laminates
Source: ACS Omega. 2025 Aug 8;10(32):36115–25. doi: 10.1021/acsomega.5c03769 (PMC12368696; doi:10.1021/acsomega.5c03769)
Supplement: Supplementary file 1 [file ao5c03769_si_001.pdf]

Supporting Information

Wettability of Kraft paper with Bio-based Impregnation Resins for the Production of High-pressure Laminates

Elisabeth Billich, [a,b] Elfriede Hogger, [a] Ivan Sumerskii, [c] Wilfried Sailer-Kronlachner, [a] Catherine Rosenfeld, [a] Martina Duller, [a] Christof Blum, [d] Antje Potthast, [b] and Hendrikus W.G. van Herwijnen, [a,d]*

[a] Wood K plus - Kompetenzzentrum Holz GmbH, Altenberger Strasse 69, 4040 Linz, Austria

[b] Department of Natural Sciences and Sustainable Resources, Institute of Chemistry of Renewable Resources, BOKU University, Konrad-Lorenz-Strasse 24, 3430 Tulln an der Donau, Austria

[c] Department of Natural Sciences and Sustainable Resources, Institute of Chemistry of Renewable Resources, Core Facility Analysis of Lignocellulosics, BOKU University, Konrad-Lorenz-Strasse 24, 3430 Tulln an der Donau, Austria

[d] Department of Natural Sciences and Sustainable Resources, Institute of Wood Technology and Renewable Materials, BOKU University, Konrad Lorenz-Strasse 24, 3430 Tulln an der Donau, Austria

* Correspondence: Department of Natural Sciences and Sustainable Resources, Institute of Wood Technology and Renewable Materials, BOKU University, Konrad Lorenz-Strasse 24,

3430 Tulln an der Donau, Austria. Wood K plus - Kompetenzzentrum Holz GmbH, Altenberger Str. 69, 4040 Linz, Austria.

E-mail addresses: erik.van-herwijnen@boku.ac.at; e.herwijnen@wood-kplus.at

Table of contents

Page S1 **Table S1.** Surface tensions of reference fluids for SFE determination.

Page S1 **Table S2.** Surface free energy of paraffin wax measured with water, diiodomethane and formamide (n=10 per fluid).

Page S1 **Table S3.** Mean contact angles of reference liquids on kraft paper surface (n=10 per fluid).

Page S2 **Table S4.** Physical properties of paper samples.

Page S2 **Figure S1.** SEM images at 250x magnification of kraft papers felt (F) and wire (W) side: VI-F (left); VI-W (right); MI-F (left); MI-W (right); RE-F (left); RE-W (right).

Page S3 **Figure S2.** Microscopy images at 10x magnification of kraft papers felt (F) and wire (W) side: VI-F (left); VI-W (right); MI-F (left); MI-W (right); RE-F (left); RE-W (right).

Page S4 **Table S5.** Carbohydrate composition of kraft paper samples determined by acidic methanolysis and GC-MS analysis.

Table S1. Surface tensions of reference fluids.

Reference fluid	γ_L (mN/m)	γ_L^p (mN/m)	γ_L^d (mN/m)
**Water**	72.8	51.0	21.8
**Diiodomethane**	50.8	0	50.8
**Formamide**	58.0	12.82	45.18

Table S2. Surface free energy of paraffin wax measured with water, diiodomethane and formamide (n=10 per fluid).

Reference Material	γ_S (mN/m)	γ_S^p (mN/m)	γ_S^d (mN/m)
Paraffin wax	22.62 (± 0.19)	0.02 (± 0.02)	22.60 (± 0.17)

Table S3. Mean contact angles of reference liquids on kraft paper surface (n=10 per fluid).

Paper type	Mean contact angle (°)		
	Water	Diiodomethane	Formamide
**VI-F**	40.11 (± 2.54)	22.52 (± 1.01)	25.56 (± 1.94)
**VI-W**	39.68 (± 2.28)	27.23 (± 1.42)	23.10 (± 2.68)
**MI-F**	51.36 (± 3.97)	32.98 (± 3.09)	32.58 (± 3.81)
**MI-W**	45.96 (± 3.96)	33.32 (± 1.82)	34.24 (± 2.30)
**RE-F**	65.91 (± 1.29)	50.53 (± 1.92)	47.28 (± 1.79)
**RE-W**	44.79 (± 4.34)	35.51 (± 2.85)	37.43 (± 2.90)

Table S4. Physical properties of paper samples.

Paper type	Thickness [mm]	Grammage [g/m^2]	Bulk [cm^3/g]	Gurley [s] (data from manufacturer)	Klemm capillary rise [mm]
**VI**	0.33 (± 0.01)	220.7 (± 6.2)	1.50 (± 0.05)	10	76 (± 1)
**MI**	0.33 (± 0.01)	223.5 (± 7.2)	1.48 (± 0.06)	11	60 (± 10)
**RE**	0.35 (± 0.01)	219.4 (± 3.5)	1.59 (± 0.03)	10	43 (± 10)

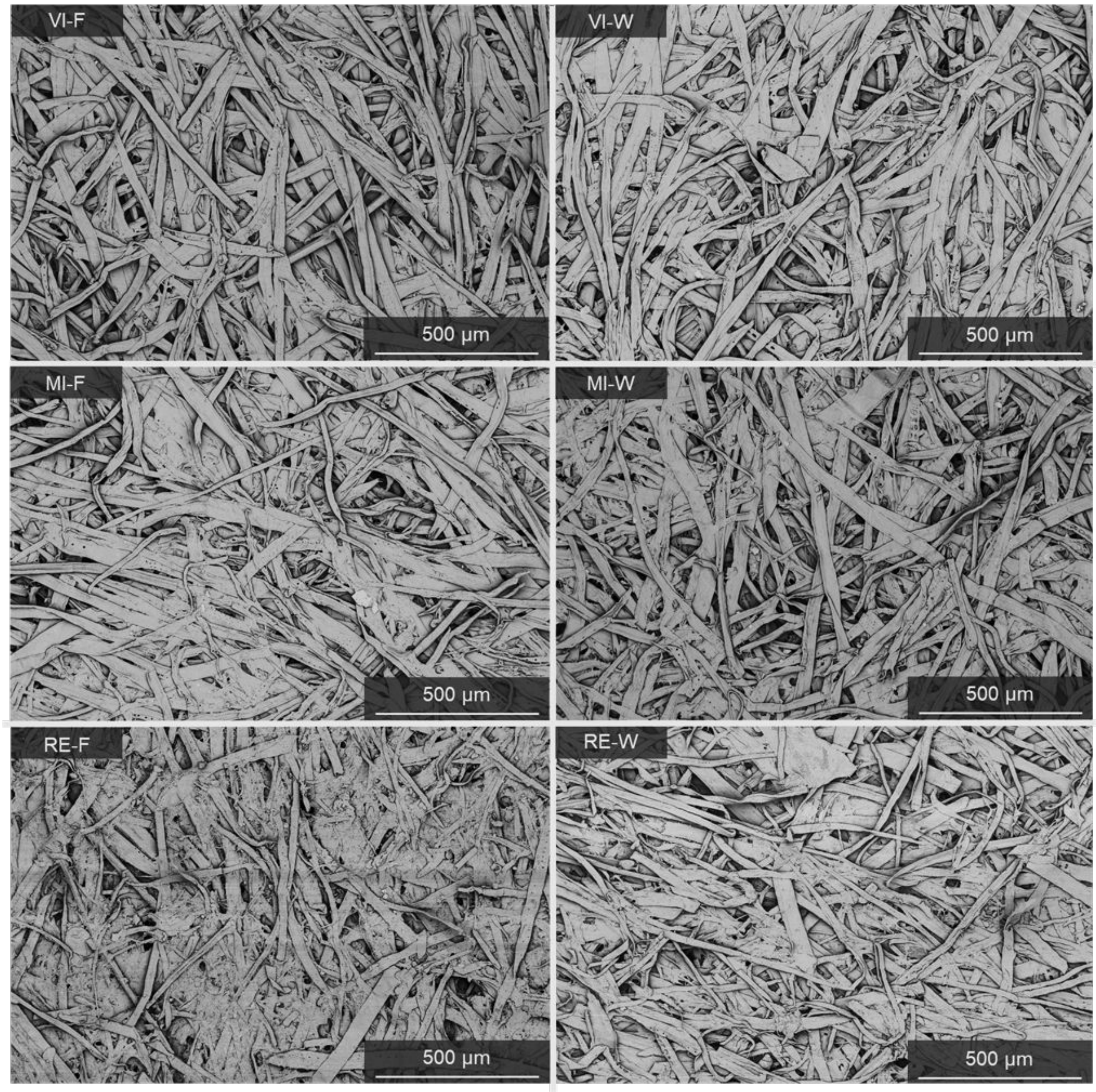

Figure S1. SEM images at 250x magnification of kraft papers felt (F) and wire (W) side: VI-F (left); VI-W (right); MI-F (left); MI-W (right); RE-F (left); RE-W (right).

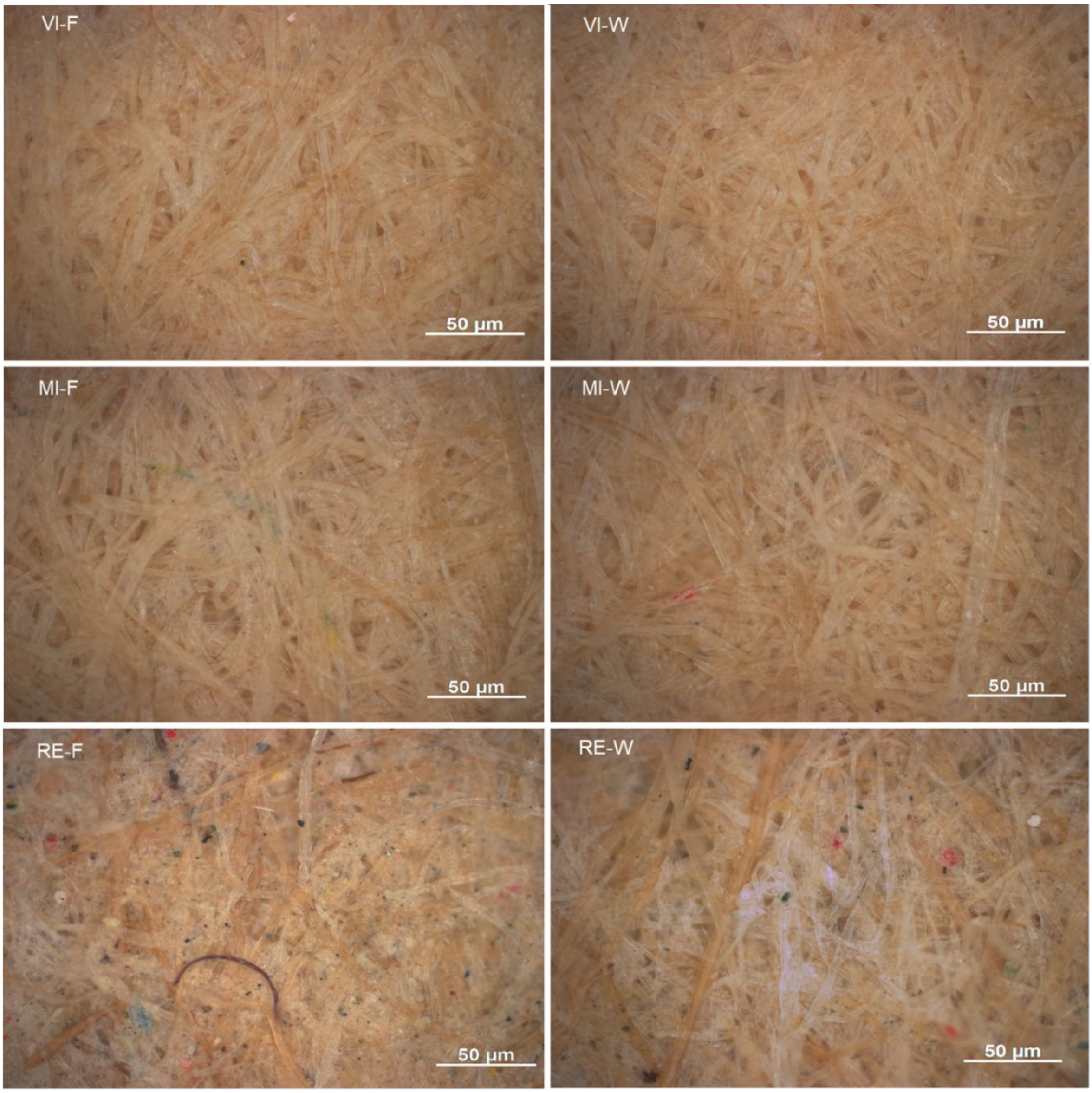

Figure S2. Microscopy images at 10x magnification of kraft papers felt (F) and wire (W) side. Top: VI-F (left); VI-W (right). Middle: MI-F (left); MI-W (right). Bottom: RE-F (left); RE-W (right).

Table S5. Carbohydrate composition of Kraft paper samples determined by acidic methanolysis and GC-MS analysis.

	**Paper Type**		
	**VI**	**MI**	**RE**
Arabinose (mg/g)	2.08 ± 0.16	2.06 ± 0.09	4.66 ± 0.09
Galactose (mg/g)	5.17 ± 0.14	4.63 ± 0.18	0.84 ± 0.07
Galacturonic acid (mg/g)	-	-	2.20 ± 0.84
Glucose (mg/g)	53.80 ± 2.00	47.03 ± 0.98	45.04 ± 1.92
Mannose (mg/g)	40.37 ± 1.39	30.67 ± 0.53	1.40 ± 0.11
4-O-Methyl-D-glucuronic acid (mg/g)	-	2.03 ± 0.20	9.56 ± 0.68
Rhamnose (mg/g)	-	0.14 ± 0.00	0.59 ± 0.02
Xylose (mg/g)	35.23 ± 1.11	35.92 ± 0.64	57.93 ± 2.30
**Total**	136.63 ± 3.93	122.48 ± 2.10	122.22 ± 5.09

References

(1) Figueiredo, A. B.; Evtuguin, D. V.; Monteiro, J.; Cardoso, E. F.; Mena, P. C.; Cruz, P. Structure−Surface Property Relationships of Kraft Papers: Implication on Impregnation with Phenol−Formaldehyde Resin. *Ind. Eng. Chem. Res.* 2011, *50* (5), 2883–2890. https://doi.org/10.1021/ie101912h.
